# Impact of Packaging Film and Beef Trimmings on Ground Beef Shelf Life

**DOI:** 10.3390/foods10081923

**Published:** 2021-08-19

**Authors:** Hunter R. Smith, Barney S. Wilborn, Anna Grace Parnell, Tristan M. Reyes, Madison P. Wagoner, Laura E. Yoder, Eugene Blythe, Don R. Mulvaney, Soren P. Rodning, Mary K. Mullenix, Tom Bonner, Jason T. Sawyer

**Affiliations:** 1 Department of Animal Sciences, Auburn University, Auburn, AL 36849, USA; hzs0101@auburn.edu (H.R.S.); wilbobs@auburn.edu (B.S.W.); agp0030@auburn.edu (A.G.P.); tzr0039@auburn.edu (T.M.R.); mpw0035@auburn.edu (M.P.W.); ley0010@auburn.edu (L.E.Y.); mulvadr@auburn.edu (D.R.M.); rodnisp@auburn.edu (S.P.R.); mullemk@auburn.edu (M.K.M.); 2 College of Agriculture, Auburn University, Auburn, AL 36849, USA; blythek@auburn.edu; 3 Winpak Ltd., 100 Saulteaux Crescent Winnipeg, Winnipeg, MB R3J3T3, Canada; tom.bonner@winpak.com

**Keywords:** color, ground beef, packaging, shelf life, TBARS

## Abstract

Fresh beef storage in the retail setting can be presented in a variety of packaging methods, and identifying an alternative such as vacuum packaging to current traditional methods could potentially increase shelf life and reduce meat waste. The objective of this study was to identify the influence of packaging film and lean trimming sources on fresh ground beef surface color during a simulated retail display period. There were no differences (*p >* 0.05) in surface color redness (a*), yellowness (b*), chroma, or hue angle regardless of packaging film or lean trimmings. However, thiobarbituric acid reactive substances (TBARS) were greater (*p <* 0.05) for packages containing a greater percentage of CULL beef trimmings regardless of packaging film. In addition, pH values of ground beef packages did not differ (*p >* 0.05) among packaging film or lean trimming blends. Visual color did not differ (*p >* 0.05) throughout the simulated retail display period regardless of beef trimmings or packaging film. Microbial spoilage organisms were greater (*p <* 0.05) after the simulated display period. These results suggest that ground beef presented in a simulated retail setting using an alternative packaging platform, such as vacuum packaging, is plausible.

## 1. Introduction

Consumer purchasing intent during the COVID-19 global pandemic resulted in excessive pressure on meat production from the farmer to retail establishments [1]. Furthermore, current fresh meat packaging in the retail setting is not designed for extended storage, forcing consumers to frequently visit retail outlets. With reductions in production capabilities of fresh beef and added limitations on purchases by retailers, a perfect storm resulted, placing a significant strain on the availability of fresh meat for the consumer [2]. Ground beef has often been a preferred product for consumers in the United States, with the sources of beef trimmings often originating from cull cows and bulls, under-valued carcass subprimals, and trimmings from the portioning (steak cutting) of beef subprimals [3].

Consumers of fresh meat products are highly influenced by surface color at the time of purchase, which is often difficult to measure in proteins, such as pork or chicken [4]. In the United States, meat, poultry, and fish account for $48 billion of food loss occurring at the retail and consumer levels annually [5]. Fresh meat has accounted for considerable food loss in the United States, at a value of $161.6 billion in 2010 [5]. Of the meat loss occurring annually, 30% has been identified at the retail counter, and 70% is lost at the consumer level [5]. With modifications in packaging technology for fresh meat and a deeper understanding of color stability in beef surface color as influenced by packaging technologies, it is plausible to reduce these annual losses that occur in the meat industry. If food loss and waste can be reduced by 50% before 2050, the world would require 1314 trillion kcals less of food per year [6]

Handling and packaging of meat products can be affected by spoilage mechanisms, such as microbial spoilage (decomposition of meat from microorganisms), lipid oxidation (also known as chemical through the degradation of lipids, proteins, and carbohydrates), and autolysis enzymatic spoilage (viewed as physical when meat becomes brittle and falls apart [7]. Vacuum-packaged beef is expected to have a shelf life of roughly 35 to 45 d at chilled temperatures [8]. An effective method identified to control meat shelf life is super chilling, where meat temperatures are stored below the initial freezing point of 1 to 2 °C, limiting the formation of ice crystals [9]. Super chilling has been considered a desirable storage method, as it allows for meat products to be stored up to four times longer than using conventional chilling [10] and storage methods.

Traditional packaging methods in the United States used by fresh meat retailers often occurs with expanded polystyrene (EPS) trays and polyvinyl chloride (PVC) film or in some instances a tray gas-flushed with a modified atmosphere (MAP). These packaging methods are intended to influence surface color and are often less likely used for extended storage in either a store retail counter, consumer refrigerator, or frozen storage application. Current fresh meat packaging methods often result in a packaged product being discarded before it is sold (sell-by date) by the retailer or eaten (use-by date) by the consumer. Investigating alternative packaging strategies, such as vacuum packaging in a form and fill system, could lend itself to greater fresh meat purchases due to extended storage in a refrigerated or frozen setting for the consumer. Several regions within the United States, such as Maine and Maryland, are implementing a ban on polystyrene containers along with major cities consisting of but not limited to New York, San Diego, Miami Beach, Seattle, and Washington, DC [11].

The objective of this experiment was to identify the influence of two vacuum packaging films (MB1 and MB2) and two sources (CULL and FED) of beef trimmings on ground beef shelf-life stability throughout a 21-day simulated retail display period.

## 2. Materials and Methods

### 2.1. Raw Materials

Fresh beef trimmings representative of FED (White Oak Pastures Inc., Bluffton, GA, USA) and CULL (Golden State Foods, Opelika, AL; USA) beef cattle carcasses were purchased from commercial meat processing facilities in the Southeast. Beef trimmings (estimated fat percentages 15 to 20%) were transported on ice in 150 qt insulated coolers (Igloo, Katy, TX, USA) to the Auburn University Lambert-Powell Meats Laboratory. The temperature of trimmings during transportation was monitored using a data recorder (ThermaData series II Temp Logger T2C, American Fork UT, USA) and downloaded upon arrival to ensure beef trimmings remained below 0 °C. Trimmings were stored in a refrigerated walk-in cooler (2 °C) in the absence of light for 24 h until grinding and packaging occurred. Beef trimmings were identified at the time of processing as FED or CULL and ground once through a 9.525-mm plate (SPECO 400, Schiller Park, IL, USA) using a commercial meat grinder (Model 4346, Hobart Corporation, Troy, OH, USA). Coarse ground beef (FED = 170.1 kg, CULL = 102.06 kg) was allocated to one of eight (Table 1) treatment batches (34.02 kg/treatment) with 3 replications (11.34 kg/replication).

Treatment batches were mixed for 2 min in a commercial meat grinder (Model 4346, Hobart Corporation, Troy, OH, USA) and finely ground once through a 3.18-mm plate (SPECO 400, Schiller Park, IL, USA). After fine grinding, ground beef was portioned into 454, ± 2 g bricks using a vacuum stuffer (VF608plus, Handtmann, Biberach, Germany), and treatments were packaged into vacuum packaging materials using a Reiser roll-stock form and fill vacuum packaging machine (Optimus OL0924, Variovac, Zarrentin, Germany). Ground beef bricks (160 mm × 135 mm ×35 mm) were packaged in a barrier film MB1 (oxygen transmission rate 0.4 cc/sq. m/24 h; vapor transmission rate 3.3 g/sq. m/24 h) or MB2 (oxygen transmission rate 0.2 cc/sq. m/24 h; vapor transmission rate 3.3 g/sq. m/24 h; WINPAK Ltd., Winipeg, MB, Canada) and placed into refrigerated tiered display cases for simulated retail display. Each treatment generated 75 ground beef packages (25 packages per replication) of ground beef.

### 2.2. Simulated Retail Display

Packages were stored in simulated retail conditions at 3 °C in a Turbo Air 3-tiered lighted retail display cooler (TOM-labels 60DXB-N, Turbo Air Inc., Long Beach, CA, USA) under continuous LED lighting. The lighting intensity of each shelf throughout the simulated display period was 2297 lux (ILT10C, International Light Technologies, Peabody, MA, USA). Storage temperatures during simulated retail display were monitored using a data-recording device (TD2F, ThermoWorks, American Fork, UT, USA) placed within the center of each shelf. Throughout the display period, refrigerated case temperatures averaged 1.94 ± 1.10 °C. In addition, ground beef packages were evenly distributed and rotated daily from side to side and front to back within the retail display cooler to eliminate temperature variation and simulate consumer package shifting at the retail counter.

### 2.3. Proximate Analysis and pH Value

Samples for proximate analysis (protein, moisture, fat, and collagen) were evaluated on the day of packaging (day 0) and subsequent measuring points throughout retail display days (7, 14, and 21). Analysis was conducted using a near-infrared (NIR) approved spectrophotometer (Food Scan™, FOSS Analytical A/S, Hilleroed, Denmark), and data processing was determined using ISIscan™ Software [12]. Ground beef pH was measured after mixing, grinding, and packaging at 7, 14, and 21 days using a pH meter (HI99163, Hanna Instruments, Woonsocket, RI, USA) equipped with a glass electrode. The pH meter was calibrated (pH 4.0 and 7.0) using 2-point standard buffers (Thermo Fisher Scientific, Chelmsford, MA, USA) prior to each sampling period and after every 10 readings.

### 2.4. Thiobarbituric Acid Reactive Substance (TBARS)

On days 0, 7, 14, and 21 of simulated retail display, ground beef was removed from the packaging material and prepared for 2-thiobarbituric acid reactive substances (TBARS) analysis using the method of Buege and Aust [13]. Approximately 4 g of ground beef was homogenized with 8 mL of cold phosphate buffer (50 mM, pH of 7.0 at 4 °C) containing 0.1% EDTA, 0.1% *n*-propyl gallate, and 2 mL trichloroacetic acid (Sigma-Aldrich, Saint Louis, MO, USA). Homogenized samples were filtered through Whatmann No. 4 filter paper, and duplicate 2-mL aliquots of the clear filtrate were transferred into 10-mL borosilicate tubes, mixed with 2 mL of 0.02 M 2-thiobarbituric acid reagent (Sigma-Aldrich, Saint Louis, MO, USA), then boiled for 20 min. After boiling, tubes were placed into an ice bath for 15 min. Absorbance was measured at 533 nm with a spectrophotometer (Turner Model–SM110245, Barnstead International, Dubuque, IA, USA) and multiplied using a factor 12.21 to obtain the TBARS value (mg malonaldehyde/kg of meat).

### 2.5. Instrumental Color Measurement

Instrumental color (L*, a*, and b*) of ground beef packages was measured through the packaging film at three different locations on each package using a HunterLab MiniScan XE Plus colorimeter, Model 45/0-L (Hunter Associates Laboratory Inc., Reston, WV, USA). Samples were read using Illuminant A, an aperture of 31.8 mm, and a 10° Observer and evaluated for CIE lightness (L*), redness (a*), and yellowness (b*) color values. Hue angle (H*) was calculated as (tan−1(b*/a*), whereas chroma (C*) was calculated as ((a*^2^ + b*^2^)^1/2^) to determine the vividness and saturation within the color space. Instrument calibration was completed prior to use on each sampling day 0, 7, 14, or 21 using black and white tiles (L*, 0 = black, 100 = white; a*, −60 = green, +60 = red; b*, −60 = blue, +60 = yellow).

### 2.6. Visual Color Evaluation

A nine-member team of expert color evaluators was used to evaluate the surface color of packaged ground beef during the simulated retail display period. Color evaluators were previously trained using [14] meat color measurement guidelines. Once daily at 1600, surface color was evaluated on day 0, 7, 14, and 21 for initial beef color (1 = light purple red; 2 = slight purple red; 3 = moderately light purple red; 4 = red; 5 = slightly dark purple; 6 = moderately dark purple red; 7 = dark purple red; and 8 = extremely dark purple red); amount of browning (1 = no evidence of browning; 2 = dull, 3 = grayish; 4 = brownish gray; 5 = brown; and 6 = dark brown); and percent (%) discoloration (1 = no discoloration (0%); 2 = slight discoloration (1 to 10%); 3 = small discoloration (11 to 25%); 4 = modest discoloration (26 to 50%); 5 = moderate discoloration (51 to 75%); 6 = extensive discoloration (76 to 99%); and 7 = total discoloration (100%)).

### 2.7. Microbial Spoilage Organisms

The total number of viable non-pathogenic aerobic microorganisms in ground beef samples was determined using standard methods [15]. Packages were opened aseptically, and 5-g samples were removed. Ground beef was placed in a stomacher bag with filter 3 M Sample Bag W/Filter Sterile (3 M Corp., St. Paul, MN, USA) with 50 mL of 3 M Butterfield’s Buffer (3 M Corp., St. Paul, MN, USA). Stomacher bags were stomached for 1 min. Samples were serially diluted using Butterfield’s buffer and plated in duplicate using Aerobic Plat Count (APC) Petrifilm^®^ (3 M Corp., St. Paul, MN, USA). Plates were incubated at 35.5 °C for 48 h in a Lab Companion incubation chamber (IB-05G, Lab Companion, Yuseong-gu, Daejeon, Republic of Korea) prior to enumeration. Counts were recorded as colony-forming units per gram (CFU/g).

### 2.8. Statistical Analysis

Data were analyzed with linear models and linear mixed models using the GLIMMIX procedure of SAS (ver. 9.4; SAS Institute Inc., Cary, NC, USA). For analysis of color ratings data, panelist was included as a random factor, and panelist x replication was included as a random, repeated factor (with a first-order autoregressive covariance structure). Fixed effects evaluated were treatment blends, packaging film, and day of display. Least-squares means were computed for all variables, and when significant (*p* ≤ 0.05) F-values were observed, least-squares means were separated using pair-wise *t*-tests (PDIFF option).

## 3. Results

### 3.1. Instrumental Analysis of Fresh Ground Beef

There was no interaction (*p* > 0.05) for instrumental fresh analysis of ground beef. However, the treatment effect on ground beef packages stored in simulated retail display conditions for thiobarbituric reactive substances (TBARs), pH, moisture, protein, fat, and collagen are presented in Table 2. There were no differences for pH (*p* > 0.05) regardless of packaging method or lean trimmings. Raw beef trimmings used for this study originated from beef carcasses that were at considered normal postmortem muscle pH ranges (5.8 to 5.3), whereas the treatment influence on TBARS values was greater (*p* < 0.05) for lean trimmings containing a greater percentage of fat. Moreover, ground beef formulations consisting of a greater percentage of CULL beef trimmings produced more (*p* < 0.05) lipid oxidation regardless of packaging materials. The moisture of ground beef packages was greater (*p* < 0.05) for packages containing a greater percentage of CULL beef trimmings. There were no differences (*p* > 0.05) for protein, fat, and collagen regardless of beef trimmings, packaging materials, or day of simulated display.

### 3.2. Fresh Beef Color

The surface color of ground beef packages stored in simulated retail display were investigated for instrumental lightness (L*), redness (a*), yellowness (b*), chroma (C*), and hue (H*) angle (Table 3). It was not surprising that the TRT blends and packaging with a greater percentage of FED trimmings and high OTR were brighter and lighter (*p* < 0.05). As fat content increased in the formulation of beef trimmings, the surface color became lighter (*p* < 0.05). However, redness (a*), vividness (C*), and hue angle (H*) were not affected by trimming formulation or packaging material (*p* > 0.05). Calculated relative values of myoglobin (deoxy, oxy, or met) using instrumental values within the spectral realm were not captured but could have elucidated additional information regarding the surface color of vacuum packaged ground beef during the simulated display period.

### 3.3. Visual Color

Visual assessment of ground beef during the simulated display for initial beef color provided no differences (*p* > 0.05) for the interaction (treatment × day) or the main effects of day or treatment (Table 4). Evaluators of surface color indicated through their assessment through the display period that surface color appeared mostly red. Surprisingly, the surface color appeared to remain stable, and it suggests that surface color in vacuum packaging could be plausible. Additional measurements using relative calculations of deoxy-, met-, and oxy-myglobin could support further visual assessment of ground beef in a vacuum package.

Treatment, day of display, nor the interaction influenced (*p* > 0.05) the visual assessment on surface browning (Table 5) throughout the simulated display. Packages of ground beef tended to have more browning appearing at the conclusion (day 21) than initially (day 0). However, evaluators indicated that the surface color of ground beef during this study appeared to have no evidence of browning. It is plausible that the OTR of the packaging materials provided protective support of the myoglobin molecule throughout the display period.

Discoloration percentages (Table 6) of surface color for vacuum-packaged ground beef was not (*p* > 0.05) influenced by lean trimmings, packaging materials nor the duration of the simulated display period. Surface color evaluators suggest that vacuum-packaged ground beef discoloration began appearing at the conclusion of the shelf-life (day 21). The lack of surface color changes suggests additional research using visual consumer panelists is necessary to further identify the acceptance of vacuum-packaged fresh meat in the retail setting. Furthermore, evaluation on the duration of shelf-life for vacuum-packaged fresh meat is necessary to determine when the surface color reaches a termination point.

### 3.4. Aerobic Changes

Analysis of ground beef packages stored in simulated retail display conditions for microbial spoilage organisms (APC) are presented in Table 7. Spoilage organisms were greater (*p* < 0.05) as the duration of the simulated display period was extended (day 14 and 21). In addition, spoilage organisms were lowest (*p* < 0.05) on d 0. It appears that as the percentage of FED trimmings increased and the percentage of CULL trimmings declined, spoilage organisms were greater (*p* < 0.05). It is plausible that the initial loading of the trimmings influenced the organisms’ growth throughout the display. Moreover, it should be noted that for the duration of the shelf-life study, there were no treatments that crossed the 6 log CFU/g threshold often considered for the wholesomeness of fresh meats.

## 4. Discussion

In this study, packaging films with varying oxygen transmission rates (OTR) from different lean trimming sources (CULL or FED) were placed into a simulated retail display setting to investigate vacuum packaging or formulation modifications for the ground beef retail consumer. Instrumental analysis of fresh surface color of vacuum-packaged ground beef was not drastically altered except for lipid oxidation (TBARS) and moisture. These limited qualitative differences are consistent with previous studies that identified several factors, such as animal diet, breed type, processing, manufacturing, logistical temperatures, and retail storage temperature conditions, which can influence surface color of meat, particularly beef, once simulated display has commenced [16,17,18]. It is plausible with the limited impact of these on vacuum-packaged ground beef that extended storage could occur.

Changes in surface color lightness (L*) values across treatments suggested that fat content likely resulted in a lighter surface color during the display period. Moreover, treatments containing more CULL trimmings likely contained a greater concentration of myoglobin, resulting in a slighter darker surface color. These color differences tend to agree with previous results where beef trimmings originating from grass-fed carcasses can contain more myoglobin, which can have greater oxidative metabolism ability [18,19,20]. Small changes in redness (a*) values across treatments in the present study were likely influenced by fat content [21,22,23] and are also similar to findings of [18,20] and due to ground beef trimming sources (grain vs. grass) finished beef trimmings. Furthermore, the lack of differences among treatments for surface color agrees with [24], where the quality grade of subprimals resulted in brighter and less discolored beef patties. In addition, the greater fat content of beef trimmings resulted in a lighter surface color due to less myoglobin, whereas beef trimmings with less fat content produced a redder surface color as a result of greater myoglobin content present in lean trimmings [25]. By reducing or eliminating surface-color changes in fresh meat, the fresh meat industry could enhance storage times and subsequently reduce the pricing reductions (markdowns) or waste (throwaways) by the retail market.

Lipid oxidation (TBARS) has been shown to increase as refrigerated storage periods are extended [24,25,26,27]. Moreover, it has been reported that a TBARS value greater than 1.0 mg maldondialdehyde/kg is a threshold for identifying off-odors in fresh meat [28]. However, more recent findings have supported TBARS values ranging from 0.6 to 2.0 mg maldondialdehyde/kg [29,30,31]. Our TBARS results are consistent with previous beef simulated shelf-life studies [16,32,33], which resulted in greater lipid oxidation (TBARs values) as storage time duration increased. Generally, increases in lipid oxidation (TBARS) over time are predominantly affected by temperature and oxygen concentration [17]. Studies have reported that vacuum-packaged meats can sustain longer display periods without adverse implications to lipid oxidation [17,33]. These studies reported that initial bacteria load coupled with temperature had a vast influence on lipid oxidation.

When consumers are selecting meat products, specifically beef, in the retail setting, they tend to place heavy emphasis on visual appearance (color) as an indicator of freshness [34,35]. Altering surface color characteristics is important to extend shelf-life of fresh meat. Interestingly, vacuum packaging is a method of storing fresh meat that provides opportunities for extending fresh storage of meat. Surface color of ground beef packaged in vacuum packaging throughout the current 21-day study declined as rated by trained evaluators. These changes in surface color are similar to other studies that note fresh meat color will eventually degrade regardless of packaging methods, antioxidant ingredient use, or storage temperature [25,36,37]. Improvements in temperature storage environment and vacuum packaging fresh meat products in the retail setting could potentially lend to minimizing markdowns and throwaways in the retail setting or by the consumer. However, additional investigation to support storage temperatures and perception of vacuum packaging use in fresh meats by consumers is warranted.

Storage temperatures, especially super-chilled storage (−1.5 °C), are noted to have the ability to inhibit the increase of bacteria load when compared to chilled storage (2 to 5 °C). However, if the initial bacteria load is excessive, then temperature becomes less of a bacteria control method [33]. Packaging materials constructed with a greater OTR are commonly used throughout the meat industry in the United States and are comprised of ethylene vinyl alcohol copolymer (EVOH) barrier, whereas a lower OTR film will be constructed with two ethylene vinyl alcohol copolymer (EVOH) barriers, preventing less oxygen transmission. Thus, it is plausible that the lower OTR film would decrease aerobic spoilage and potentially extend shelf life by mitigating lipid oxidation and microbial spoilage growth. Spoilage organisms in the current study were greater at the conclusion of a 21-day shelf-life than initially on day 0. These findings tend to agree with previous research [35], where focused efforts on packaging OTR was reported to be an influencer of aerobic spoilage. It appears that as barrier properties improve limiting oxygen transmission, spoilage organism growth can be altered.

The creativity of vacuum packaging for ground beef allows for the product to be placed into a pouch or molded form and the atmosphere extracted from the package using a vacuum machine. With fresh meat color dependance on oxygen for the bright-cherry red appearance, vacuum packaging can inhibit the surface-color brightness due to a lack of oxygen. Changes to the surface color of fresh meat can impact consumer purchasing decisions at the retail counter [38]. The advances in packaging technologies as described in the current study could provide an alternative method for reducing the losses that occur due to ground beef surface discoloration [39].

## 5. Conclusions

The use of vacuum packaging through a form and fill system (roll-stock) for ground beef could be a viable future option for use in the retail setting. The current results suggest that the fresh color properties of ground beef under these packaging types can withstand extended storage periods (up to 21 days) in a simulated retail setting. Changes to qualitative and surface color characteristics were minimal and suggest that neither source of lean trimmings nor packaging caused detrimental changes to the ground beef during a simulated retail display period.

Meat consumers remain committed to placing a tremendous emphasis on surface color, focused mostly on the redness of the meat surface at the time of retail purchases. The current results support surface redness of vacuum-packaged ground beef was not impacted. Furthermore, it is promising that, within the current study, vacuum packaging is a plausible option for ground beef platforms in the retail setting for extending fresh beef shelf life up to 21 days. With limited changes to the surface color of fresh ground beef during a 21-day shelf life, it would be beneficial to identify additional research to further elucidate the termination point (shelf life) of fresh, vacuum-packaged ground beef. In addition, new research would be beneficial to identify additional changes that could occur to sensory factors of taste and visual characteristics by consumer panelists before the adoption of vacuum packaging of fresh beef throughout the fresh meat retail setting.

## Figures and Tables

**Table 1:** Assignment of treatment identifier with beef trimmings and packaging films.

Treatment	Packaging Film ^1^	CULL Trimmings ^2^	FED Trimmings ^3^
TRT 1	MB1	75	25
TRT 2	MB1	50	50
TRT 3	MB1	25	75
TRT 4	MB1	0	100
TRT 5	MB2	75	25
TRT 6	MB2	50	50
TRT 7	MB2	25	75
TRT 8	MB2	0	100

^1^ Packaging film oxygen transmission rate (OTR) for MB1 (0.4 cc/sq. m/24 h); MB2 (0.2 cc/sq. m/24 h); vapor transmission rate (VTR) of (3.3 g/sq. m/24 h) and film thickness (7 mm). ^2^ Percentage (%) of CULL trimmings used in the formulation of ground beef originated from cull bull and cow beef carcasses. ^3^ Percentage (%) of FED trimmings used in the formulation of ground beef originated from fed beef steer and heifer beef carcasses.

**Table 2:** Influence of packaging film and lean trimmings on instrumental analysis of ground beef.

	Treatment								
	TRT 1	TRT 2	TRT 3	TRT 4	TRT 5	TRT 6	TRT 7	TRT 8	SEM *
pH	5.47	5.53	5.61	5.59	5.49	5.56	5.56	5.60	0.012
TBARS ^1^	1.46 ^a^	1.34 ^b^	1.31 ^bc^	1.22 ^c^	1.40 ^ab^	1.37 ^ab^	1.29 ^bc^	1.30 ^bc^	0.028
Moisture ^2^	68.18 ^b^	68.06 ^b^	69.61 ^a^	69.79 ^a^	68.50 ^b^	67.85 ^b^	70.04 ^a^	69.84 ^a^	0.017
Protein ^3^	21.42	22.15	23.12	22.62	21.87	22.28	22.65	22.73	0.015
Fat ^4^	15.97	15.82	13.49	12.98	15.81	16.32	13.51	12.76	0.109
Collagen ^5^	4.77	4.94	4.62	3.95	5.27	5.08	4.55	4.04	0.091

^1^ 2-Thiobarbituric acid reactive substances (TBARS) are a measure of lipid oxidation, with a larger value indicating a greater amount of oxidation (mg malonaldehyde/kg of tissue). ^2^ Moisture percentage (g/100 g). ^3^ Protein percentage (g/100 g).^4^ Fat percentage (g/100 g).^5^ Collagen percentage (g/100 g). * SEM, standard error of the mean. ^a–c^ Mean values within a row lacking common superscripts are significantly different (*p <* 0.05).

**Table 3:** Influence of packaging film and lean trimmings on instrumental fresh surface color of ground beef during a simulated retail display shelf life.

	Treatment								
	TRT 1	TRT 2	TRT 3	TRT 4	TRT 5	TRT 6	TRT 7	TRT 8	SEM *
L* ^1^	46.44 ^ab^	45.27 ^b^	43.99 ^d^	42.40 ^e^	46.99 ^a^	46.18 ^c^	43.56 ^d^	41.97 ^e^	0.206
a* ^2^	21.65	22.63	22.69	23.55	21.12	23.37	23.01	23.11	0.661
b* ^3^	13.88	14.07	13.58	13.67	14.10	13.64	13.90	12.98	0.076
Chroma (C*) ^4^	25.77	26.68	26.46	27.23	25.51	25.55	26.91	26.51	0.108
Hue Angle (°) ^5^	32.86	32.00	30.97	30.17	34.05	32.61	31.32	29.37	0.149

^1^ L* (lightness) values are a measure of darkness to lightness (larger value indicates a lighter color; 100 is white, and 0 is black). ^2^ a* (redness) values are a measure of redness (larger value indicates a redder color; +60 is red, and −60 is green). ^3^ b * (yellowness) values are a measure of yellowness (larger value indicates a more yellow color; +60 is yellow, and −60 is blue). ^4^ Chroma (C*) is a measure of total color (a larger number indicates a more vivid color). ^5^ Hue angle (H*) represents the change from the true red axis (a larger number indicates a greater shift from red to yellow). * SEM, standard error of the mean. ^a–e^ Mean values within a row lacking common superscripts are significantly different (*p <* 0.05).

**Table 4:** Influence of packaging film and lean trimmings on initial beef color evaluator ratings of ground beef during a simulated retail display shelf life.

	Treatment								
	TRT 1	TRT 2	TRT 3	TRT 4	TRT 5	TRT 6	TRT 7	TRT 8	SEM *
DAY 0	4.11	4.45	4.44	4.71	4.18	4.06	4.36	4.70	0.319
DAY 7	4.15	4.15	4.28	4.50	4.03	4.24	4.39	4.68	0.328
DAY 14	4.36	4.36	4.35	4.67	4.30	4.59	4.42	4.77	0.319
DAY 21	4.12	4.24	4.49	4.70	4.24	4.44	4.40	4.38	0.328

Initial beef color (1 = light purple red; 2 = slight purple red; 3 = moderately light purple red; 4 = red; 5 = slightly dark purple; 6 = moderately dark purple red; 7 = dark purple red; and 8 = extremely dark purple red). * SEM, standard error of the mean.

**Table 5:** Influence of packaging film and lean trimmings on amount of browning color evaluator ratings of ground beef during a simulated retail display shelf life.

	Treatment								
	TRT 1	TRT 2	TRT 3	TRT 4	TRT 5	TRT 6	TRT 7	TRT 8	SEM *
DAY 0	1.27	1.18	1.06	1.00	1.30	1.36	1.15	1.03	0.114
DAY 7	1.27	1.41	1.23	1.05	1.32	1.16	1.07	1.02	0.121
DAY 14	1.27	1.15	1.21	1.15	1.30	1.27	1.15	1.15	0.115
DAY 21	1.64	1.52	1.62	1.45	1.75	1.47	1.60	1.35	0.121

Amount of browning (1 = no evidence of browning; 2 = dull; 3 = grayish; 4 = brownish gray; 5 = brown; and 6 = dark brown). * SEM, standard error of the mean.

**Table 6:** Influence of packaging film and lean trimmings on percent discoloration color evaluator ratings of ground beef during a simulated retail display shelf life.

	Treatment								
	TRT 1	TRT 2	TRT 3	TRT 4	TRT 5	TRT 6	TRT 7	TRT 8	SEM *
DAY 0	1.00	1.00	1.00	1.00	1.03	1.03	1.00	1.00	0.096
DAY 7	1.06	1.29	1.10	1.00	1.23	1.10	1.08	1.07	0.102
DAY 14	1.27	1.21	1.18	1.15	1.24	1.30	1.12	1.12	0.096
DAY 21	1.54	1.53	1.51	1.41	1.75	1.47	1.58	1.36	0.101

Percent (%) discoloration (1 = no discoloration (0%); 2 = slight discoloration (1 to 10%); 3 = small discoloration (11 to 25%); 4 = modest discoloration (26 to 50%); 5 = moderate discoloration (51 to 75%); 6 = extensive discoloration (76 to 99%); and 7 = total discoloration (100%). * SEM, standard error of the mean.

**Table 7:** Interactive influence of treatment × day of display on microbial spoilage organisms (APC) of ground beef during a simulated retail display.

	Treatment								
	TRT 1	TRT 2	TRT 3	TRT 4	TRT 5	TRT 6	TRT 7	TRT 8	SEM *
DAY 0	1.20 ^cd^	1.69 ^ab^	1.32 ^bcd^	1.94 ^a^	0.96 ^d^	1.26 ^bcd^	1.37 ^bcd^	1.42 ^bc^	0.054
DAY 7	2.55 ^ab^	2.98 ^a^	2.37 ^b^	2.60 ^ab^	2.66 ^ab^	2.31 ^b^	2.65 ^ab^	2.32 ^b^	0.054
DAY 14	2.92 ^ab^	2.62 ^bc^	2.42 ^c^	2.32 ^c^	3.11 ^a^	2.52 ^bc^	2.38 ^c^	2.30 ^c^	0.077
DAY 21	2.64 ^bcd^	2.75 ^bc^	3.24 ^a^	2.20 ^e^	2.59 ^bcde^	2.42 ^cde^	3.02 ^ab^	2.31 ^de^	0.054

APC Log colony forming units per gram (CFU/g or Log CFU/cm^2^) of sampled ground beef. * SEM, standard error of the mean. ^a–e^ Mean values lacking common superscripts are significantly different (*p <* 0.05).
